# Acute aerobic exercise enhances associative learning in regular exercisers but not in non-regular exercisers

**DOI:** 10.3389/fnbeh.2024.1515682

**Published:** 2025-01-07

**Authors:** Kayleigh D. Gultig, Cornelis P. Boele, Lotte E. M. Roggeveen, Ting Fang Soong, Sebastiaan K. E. Koekkoek, Chris I. De Zeeuw, Henk-Jan Boele

**Affiliations:** ^1^Department of Neuroscience, Erasmus MC, Rotterdam, Netherlands; ^2^BlinkLab Ltd., Sydney, NSW, Australia; ^3^Department of Neuroscience, Vrije Universiteit, Amsterdam, Netherlands; ^4^Netherlands Institute for Neuroscience, Royal Academy of Arts and Sciences, Amsterdam, Netherlands; ^5^Neuroscience Institute, Princeton University, Princeton, NJ, United States

**Keywords:** aerobic exercise, acute exercise, learning, lifestyle, neurophysiology, eyeblink conditioning

## Abstract

**Introduction:**

Physical exercise has repeatedly been reported to have advantageous effects on brain functions, including learning and memory formation. However, objective tools to measure such effects are often lacking. Eyeblink conditioning is a well-characterized method for studying the neural basis of associative learning. As such, this paradigm has potential as a tool to assess to what extent exercise affects one of the most basic forms of learning. Until recently, however, using this paradigm for testing human subjects in their daily life was technically challenging. As a consequence, no studies have investigated how exercise affects eyeblink conditioning in humans. Here we hypothesize that acute aerobic exercise is associated with improved performance in eyeblink conditioning. Furthermore, we explored whether the effects of exercise differed for people engaging in regular exercise versus those who were not.

**Methods:**

We conducted a case–control study using a smartphone-based platform for conducting neurometric eyeblink conditioning in healthy adults aged between 18 and 40 years (*n* = 36). Groups were matched on age, sex, and education level. Our primary outcome measures included the amplitude and timing of conditioned eyelid responses over the course of eyeblink training. As a secondary measure, we studied the amplitude of the unconditioned responses.

**Results:**

Acute exercise significantly enhanced the acquisition of conditioned eyelid responses; however, this effect was only true for regularly exercising individuals. No statistically significant effects were established for timing of the conditioned responses and amplitude of the unconditioned responses.

**Discussion:**

This study highlights a facilitative role of acute aerobic exercise in associative learning and emphasizes the importance of accounting for long-term exercise habits when investigating the acute effects of exercise on brain functioning.

## 1 Introduction

Physical exercise is often proposed to have beneficial effects on brain function, including learning and memory formation (Perini et al., 2016; Winter et al., 2007). Exercise is defined as a subcategory of physical activity encompassing intentional physical activity that is planned, structured and repetitive (Caspersen et al., 1985). The reported short- and long-term effects of exercise on the brain are, however, variable (Basso and Suzuki, 2017; Chang et al., 2012; Loprinzi et al., 2021), with some suggesting the benefits to be exaggerated (Ciria et al., 2023). A more objective way to investigate if and how exercise impacts learning is through Pavlovian eyeblink conditioning, a well-established paradigm to study associative motor learning. In eyeblink conditioning, an unconditional stimulus (US) that reliably evokes a reflexive eyeblink, is repeatedly paired with a conditioned stimulus (CS). Eventually the CS itself will evoke an anticipatory eyeblink, which is called a conditioned response (CR) (Marcos Malmierca and Marcos de Vega, 2017). The neural circuits and plasticity mechanisms underlying eyeblink conditioning have been studied extensively in both experimental animals and human participants. In mice, acute exercise during eyeblink conditioning, such as voluntary or externally imposed treadmill running, enhances learning and expression of conditioned eyelid responses (Albergaria et al., 2018; Broersen et al., 2023). Furthermore, short-term voluntary wheel running enhanced delay eyeblink conditioning in rats (Green et al., 2011) and, in conjunction with environmental enrichment, reversed alcohol-induced trace eyeblink conditioning deficits in rats (Hamilton et al., 2014). However, chronic physical exercise in the form of a physically enriched environment does not seem to improve learning or CR expression in rodents (Dijkhuizen et al., 2024), though it has small but significant effects on the adaptive timing of CRs. To our knowledge, the effects of exercise on eyeblink conditioning in humans have not yet been investigated.

In this exploratory study, we examine the effects of acute aerobic exercise on cerebellar associative learning in healthy adults, using a smartphone-based platform to conduct eyeblink conditioning tests. We assess the effects of acute exercise in individuals who engage in regular exercise compared to those who do not. Based on the reported acute effects of physical exercise in mice, and the fact that eyeblink conditioning mechanisms are conserved across species (Steinmetz et al., 2001), we hypothesize that aerobic exercise will similarly facilitate eyeblink conditioning in humans. Furthermore, since long-term exercise influences the brain’s response to acute exercise (Basso and Suzuki, 2017), we expect that the exercise-enhancing effects on eyeblink conditioning will be greater in regularly active individuals compared to more sedentary participants.

## 2 Materials and methods

### 2.1 Participants

Forty neurotypical participants aged between 18 and 40 years, were recruited by social media invitations to participate in the study. Based on a previous eyeblink conditioning study in humans using the same protocol (Boele et al., 2023), a sample size of four participants per group (*α* = 0.05, standardized mean difference effect size = 2.71, power = 0.95) was determined as sufficient to detect an effect of session on conditioned response amplitude. Furthermore, the sample size in this pilot study is in-line with other eyeblink conditioning research in humans (Kimpel et al., 2020; Löwgren et al., 2017). Participants were divided into an active or sedentary lifestyle group based on their average weekly hours of exercise. Weekly hours of moderate and vigorous exercise were established by asking two questions about weekly exercise adapted from the short form of International Physical Activity Questionnaire (IPAQ). This IPAQ consists of seven questions and is a validated tool for monitoring physical activity levels in diverse adult populations aged between 18 and 65 years (Craig et al., 2003). We based our questions about participants’ weekly exercise on the four IPAQ questions most relevant to exercise. The cut-off point for active or sedentary group classification was determined using the lower limit of the WHO guidelines for physical activity in adults aged 18–64 years (World Health Organization, 2020). Participants doing less than 2.5 h of moderate intensity or less than 75 min of vigorous intensity exercise were in the sedentary group and the other participants were in the active group. Thus active and sedentary in this study refer to pre-existing exercise levels rather than more general levels of physical activity. Moderate intensity was defined as: “Exercise that increases heart rate but you are still able to hold a conversation” and vigorous as “Exercise that raises your heart rate so that you are unable to speak.” Education level was similar across groups as all subjects either had a university degree or were university students. Furthermore, the average age and hours of sleep per night were similar across groups (Table 1). Exclusion criteria included: current or previous neurological, neurodevelopmental or neuropsychiatric condition; current use of medication affecting the central nervous system, acute infection; current or previous known cardiovascular condition or obesity. This study was approved by the Institutional Review Board for Human Subjects of Princeton University (IRB #13943). All participants were informed of the study protocol and gave their written informed consent.

**Table 1:** Demographic and exercise-related data for participants in the active and sedentary lifestyle groups with or without an exercise intervention.

	Active, post-exercise (*n* = 9)	Active, no-exercise (*n* = 9)	Sedentary, post-exercise (*n* = 9)	Sedentary, no-exercise (*n* = 9)	Statistical test	*p*-value
	Mean (±SD)					
Moderate exercise (hours/week)	3.2 (±1.6)	4.7 (±2.4)	0.9 (±0.8)	1.1 (±0.8)	*F*_3, 32_ = 12.5	<0.0001
Vigorous exercise (hours/week)	4.1 (±3.1)	2.1 (±1.3)	0.2 (±0.3)	0.0 (±0.0)	*F*_3, 29_ = 9.6	0.0001
Total exercise (hours/week)	7.3 (±3.4)	6.8 (±2.9)	1.1 (±0.8)	1.1 (±0.8)	*F*_3, 32_ = 20.3	<0.0001
Age (years)	24.9 (±2.3)	25.6 (±5.9)	22.2 (±3.6)	24.9 (±5.0)	*F*_3, 32_ = 1.01	0.40
Sleep (hours/night)	7.5 (±0.8)	7.9 (±0.3)	7.6 (±1.2)	7.3 (±0.4)	*F*_3, 30_ = 0.8	0.49
Days between EBC sessions	0.8(±0.5)	0.8(±0.7)	1.2(±1.7)	1.1(0.9)	H3 = 0.8	0.84
Average HR during exercise (bpm)	143.7 (±18.6)	–	152.4 (±20.0)	–	*t*_11_ = −0.8	0.43
Average duration of exercise intervention (minutes)	33.5 (±9.6)	–	31.0 (±3.6)	–	*Z* = −0.3	0.82
Self-rated exercise intensity /5	2.8 (±0.5)	–	3.7 (±1.0)	–	*t*_11,51_ = −2.3	0.04
**Sex**						
Male	4 (44.4%)	4 (44.4%)	3 (33.3%)	5 (55.6%)	–	–
Female	5 (55.6%)	5 (55.6%)	6 (66.7%)	4 (44.4%)	–	–

Statistical comparisons done using one-way ANOVAs for exercise, age and sleep; a *t*-test for unequal variances for exercise intensity ratings, Kruskal-Wallis test for days between EBC sessions, a *t*-test for average heart rate and Wilcoxon’s rank sum test for average exercise intervention duration. SD, standard deviation; EBC, eyeblink conditioning; HR, heart rate; bpm, beats per minute.

### 2.2 Experiments

**Figure 1:**
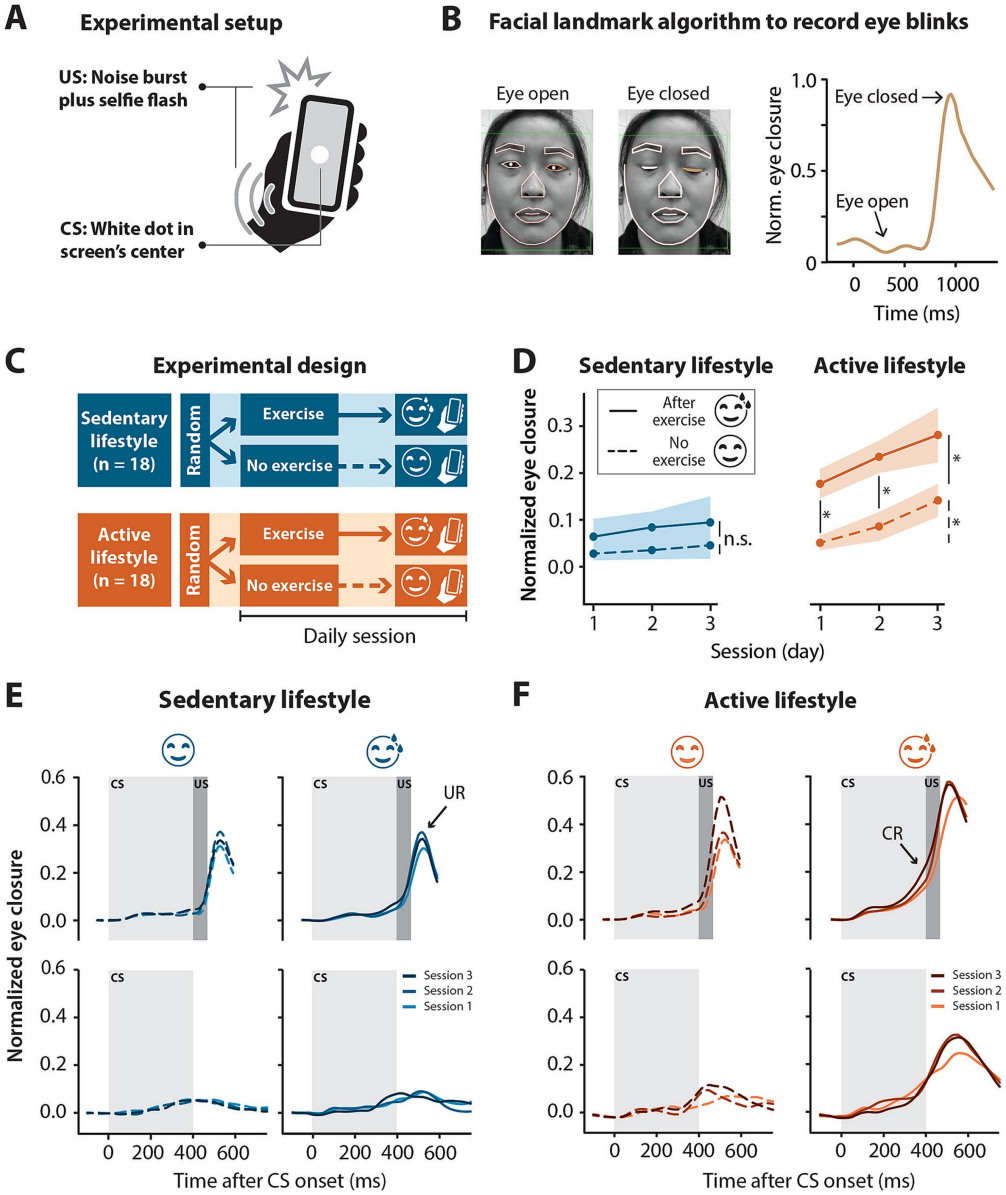
Smartphone-mediated eyeblink conditioning in participants with an active versus sedentary lifestyle. Half exercised prior to the conditioning sessions and the other half did not. **(A)** Experimental setup for smartphone-mediated eyeblink conditioning. The conditioned stimulus (CS) was a 1 cm diameter white circle presented in the center of the screen for 450 ms. The unconditioned stimulus (US) was a simultaneous 50 ms full screen flash and 105 dB 50 ms white noise pulse, presented at 400 ms and co-terminating with the CS at 450 ms. **(B)** Facial landmark detection algorithm detects eyelid movements recorded by the smartphone’s forward facing camera in real-time. In this example, the raw signal amplitude is recorded from the left eye. A value of 0 corresponds to eye open and a value of 1 corresponds to eye closed. **(C)** Illustration of experimental design. Sedentary (<2.5 h of exercise in a week) and active (>2.5 h of exercise in a week) individuals were randomly assigned to a no-exercise or an exercise intervention. The exercise intervention completed three eyeblink conditioning sessions directly after moderate intensity running or cycling whereas the no-exercise intervention did no exercise for at least 8 h prior to the three eyeblink conditioning sessions. No more than one session was done on a day. **(D)** Normalized eye closure amplitude by session for all paired (CS + US) and CS-only trials combined (regardless of eye closure amplitude) in the sedentary and active groups with or without an exercise intervention before eyeblink conditioning sessions. Active individuals showed significant conditioning with the post-exercise intervention showing significantly higher conditioned response amplitudes at sessions 1 and 2 compared to the no exercise intervention. Colored shading represents standard error of the mean. **(E)** Sedentary and **(F)** active group averaged eyelid traces for paired (CS + US) trials (top panels) and CS-only trials (bottom panels) without (left panels) or after (right panels) exercise for three eyeblink conditioning sessions. Light gray blocks indicate the presentation of the CS for 450 ms and dark gray blocks indicate the presentation of the US for 50 ms co-terminating with the CS at 450 ms. In paired trials, note the peak in amplitude following the presentation of the US, namely the unconditioned response (UR) present in all groups regardless of the intervention. Note the shift in timing of the rise in amplitude in paired trials to precede the presentation of the US at later sessions—conditioned response (CR)—especially obvious in the active, post-exercise intervention. The acquisition of conditioned responses over the three sessions is also illustrated by the rise in amplitude in the CS-only trials, again particularly obvious in the active, post-exercise intervention. Significance levels: *p < 0.05, n.s. = not significant.

Experiments were conducted via the BlinkLab smartphone application (Boele et al., 2023). During the experiment, participants watched audio-normalized nature documentaries or TV shows. A delay eyeblink conditioning paradigm, a form of cerebellar associative learning (Albergaria et al., 2018; Thompson, 1983), was used in this study. The eyeblink conditioning experiment consisted of the pairing of a CS with a US (previously described by Boele et al., 2023; Figures 1A,B). It was previously shown that eyeblink conditioning conducted with the BlinkLab application produced conditioned responses comparable to responses obtained with traditional eyeblink conditioning methods (Boele et al., 2023). The CS, a circular white dot 1 cm in diameter, was presented in the center of the phone screen for 450 ms. The US was a simultaneous full-screen flash and 105 dB white noise pulse presented for 50 ms. While different from the traditional airpuff US, a non-somatosensory US, such as the US in our study, has been successfully implemented in both animal (Rogers et al., 1999) and human (Marcos Malmierca and Marcos de Vega, 2017) studies. In paired trials, the US was presented 400 ms after the onset of the CS and co-terminated with the CS. In US-only trials, the stimuli were presented for 50 ms, 400 ms from trial onset. Each eyeblink conditioning session consisted of 10 blocks. Within each block, there were 8 paired trials, 1 CS-only trial, 1 US-only trial and 6 “dummy” trials, no stimuli were presented but eyelid data was recorded for 6 s, semi-randomly distributed throughout the block.

### 2.3 Procedure

#### 2.3.1 Experimental setup

Participants were instructed to use headphones and complete the experiments in a quiet, well-lit room either at their place of residence or another quiet room easily accessible following their exercise. As previously reported the application continuously monitors the environment to ensure an adequate testing environment for the remote experiment (Boele et al., 2023). All participants completed three sessions of experiments in the space of a week, with no sessions done on the same day. Groups did not differ significantly in the mean interval between eyeblink conditioning sessions (Table 1).

#### 2.3.2 Exercise groups

Participants in the active and sedentary groups were randomly assigned to an exercise and no-exercise “intervention.” Participants in the exercise intervention were instructed to do all eyeblink conditioning sessions as soon as possible after at least 30 min of moderate intensity running or cycling and to record the activity using a smartwatch if they had access to such a device. Subjective monitoring of exercise intensity was done by the participants who, based on the linear relationship between exercise intensity and ventilation that occurs when exercise is below the ventilatory threshold (Goode et al., 1998; Reed and Pipe, 2014), were instructed to continue exercising at an intensity level where they could hold a conversation.

Participants in the no-exercise intervention were instructed to refrain from exercise for at least 8 h before the test (Figure 1C). Before starting the eyeblink training session, participants were asked, in the app, to rate the intensity of the exercise on a five-point Likert type scale (Likert, 1932) (Table 1).

### 2.4 Data processing

Data processing was done in R 4.3.1. Trials were baseline corrected using the 500 ms stimulus-free baseline and min-max normalized using spontaneous blinks as a reference. Individual eyelid traces were normalized by dividing each trace by the maximum signal amplitude of the relevant session. Thus, eyes closed corresponded to a value of 1 and eyes open to a value of 0 (Figure 1B) (Boele et al., 2023).

Trials with extreme outliers (signal amplitude < −0.4) and trials where spontaneous blinks occurred within a time window of 150 ms before, until 35 ms after stimulus presentation were excluded from further analysis. Trials were then re-baseline corrected using the same time window that was used for removal of spontaneous blinks.

CR amplitude was determined as the maximum signal amplitude value at 430 ms, for paired and CS-only trials. This time value was chosen to allow for a latency of 30 ms following the expected presentation of the US at 400 ms. There is a latency in response to the US (Supplementary Figure S1) likely due to retinal processing of the flash (Rogers et al., 1999).

In this study we did not include an explicitly unpaired control group to account for pseudoconditioning, however, we did analyze spontaneous blinks over the course of conditioning. During the “dummy” trials in each experiment, 6s of eyelid data was recorded and captured in the absence of stimuli delivery. Spontaneous blinks were defined as peaks in the eyelid signal data with an amplitude >0.5. The number of spontaneous blinks per second were determined for each subject for every session and then compared between exercise vs. no exercise conditions within active and sedentary groups.

To compare latency to CR peak between groups, CS-only trials were analyzed. Here, CRs were defined as trials with a maximum signal amplitude above 0.10 in a time window ranging from 60 to 750 ms. Additionally, the mean percentage of well-timed CRs was calculated per group. A well-timed CR was defined as a trial with a maximum signal amplitude above 0.10 in a time window between 400 and 500 ms.

### 2.5 Statistical analysis

All statistical analyzes and visualizations were done in R 4.3.1. Prior to comparing potential differences in variables between groups, the Shapiro–Wilk test (Shapiro and Wilk, 1965) was used to assess normality and Levene’s test (Levene, 1960) was used to assess equality of variances. If the Shapiro–Wilk test was significant (*p* < 0.05), non-parametric tests were used to compare groups, else parametric tests were used. If Levene’s test was significant (*p* < 0.05), statistical tests that allow for unequal variances were used, else statistical tests assuming equal variances were used. Potential differences between groups in age, average weekly exercise and sleep hours were tested using a one-way ANOVA. A *t*-test for unequal variances was used to compare the self-reported exercise intensity levels between the active and sedentary groups who completed eyeblink conditioning after exercise. Average heart rate was compared between active and sedentary post-exercise groups using a *t*-test. Wilcoxon’s rank sum test was used to compare average exercise duration for the active and sedentary groups post-exercise intervention. Potential differences in mean duration between EBC sessions were tested using the Kruskal-Wallis test.

For all other analyzes, multilevel linear mixed effects (LME) models were used. These models are robust to deviations from normality and are more appropriate for the nested data structure of this study (Aarts et al., 2014; Schielzeth et al., 2020). In all models, “subject” was used as a random effect. For CR amplitude models, a random slope for the effect of sessions across subjects was used. Model assumptions were visually inspected using the plot_model function from the R package sjPlot. Clear deviations from homoscedasticity and normal distribution of the residuals were observed in the CR amplitude models but not for any other models. Thus, data for the CR amplitude models was normalized using ordered quantile normalization, suggested by the bestNormalize package in R, to allow for optimal model fit. Fixed effects included: “session,” “exercise,” and “exercise*session.” The same fixed effects were used in the LME model evaluating spontaneous blinks. Fixed effects for the latency to CR peak models included: “exercise” and “CR amplitude.” The models for well-timed CRs had “exercise” as a fixed effect. The restricted maximum likelihood method was used to estimate model parameters. Log likelihood ratio and AIC and BIC indices were used to assess the model fit. An alpha value of *p* < 0.05 (two-tailed) was used to determine significance. For multiple comparisons, Bonferroni-Holm *p*-value adjustments were made to account for the number of comparisons.

## 3 Results

### 3.1 Participant overview

A total of 40 neurotypical participants were initially included in the study, which was conducted over the period from 28 December 2022 until 31 May 2023. Three participants were excluded during data pre-processing due to hardware-related latency issues. One participant in the active, post-exercise group was excluded due to a complete lack of eyeblink startle responses in all sessions; four sessions from different participants were excluded due to Wifi-related technical issues with the application: session 1 for one participant in the sedentary, post-exercise intervention; session 1 for one participant in the active, no-exercise intervention; session 2 for one participant in the active, no-exercise intervention and session 3 for one participant in the active, no-exercise intervention. The final cohort included 18 individuals in each lifestyle group, split into nine individuals per intervention (exercise vs. no exercise) (Table 1). The total, moderate and vigorous hours of exercise per week differed significantly between the active and sedentary groups (Table 1). All mean values presented below are ± standard deviation (SD) and *p*-values are Bonferroni-Holm corrected for multiple comparisons.

### 3.2 Exercise

Participants in the sedentary, post-exercise intervention completed all three sessions on average 11 min following exercise while those in the active, post-exercise intervention completed all three sessions on average 14 min following exercise. Two participants in the sedentary post-exercise intervention and two in the active post-exercise intervention did not perform the instructed exercise type for one of the three sessions. The frequency of exercise types completed prior to eyeblink conditioning can be seen in Supplementary Figure S2. The average duration of exercise completed prior to eyeblink conditioning can be seen in Table 1 and did not differ significantly between the active and sedentary groups (*Z* = −0.3, *p* = 0.82). Two participants in the sedentary post-exercise intervention and one in the active post-exercise intervention were not able to record their heart rate with a smartwatch. Average heart rate data for the remaining participants can be seen in Table 1 and did not differ significantly between active and sedentary participants (*t*_11_ = −0.8, *p* = 0.43).

Both active and sedentary individuals in the no-exercise intervention completed all three sessions without any aerobic exercise for at least 8 h before the test.

### 3.3 Conditioning—acquisition

While some participants did not acquire CRs (Supplementary Figure S3A), in others the acquisition of CRs already started to occur in session 1 with the amplitude and timing of these responses improving over the course of three sessions, pointing toward associative learning rather than pseudoconditioning (Supplementary Figure S3B).

In order to assess the effect of aerobic exercise on eyeblink conditioning we compared the CR amplitude between no and post-exercise interventions within the sedentary and active groups separately. For the sedentary group, CR amplitudes in the no exercise intervention were low (session 1 mean = 0.03 ± 0.15, Table 2) and did not really increase by session 3 (mean = 0.05 ± 0.18). In the post-exercise intervention, CR amplitudes were slightly higher (session 1 mean = 0.07 ± 0.20) and showed a slight increase by session 3 (mean = 0.10 ± 0.23). Within the sedentary group, no main effect of session (*F*_2, 3,476_ = 1.41, *p* = 0.24) or exercise (*F*_1, 16_ = 1.49, *p* = 0.24) was found and there was no significant interaction between session and exercise (*F*_2, 3,476_ = 0.78, *p* = 0.46) (Figures 1D,E; Supplementary Table S1).

**Table 2:** Conditioned response amplitudes, latencies and spontaneous blinks in active and sedentary groups with and without an exercise intervention.

	Sedentary no-exercise		Sedentary post-exercise		Active no-exercise		Active post-exercise	
Session	Mean	SD	Mean	SD	Mean	SD	Mean	SD
**CR amplitude (mean NEC)**								
1	0.03	0.15	0.07	0.20	0.05	0.15	0.18	0.27
2	0.04	0.16	0.09	0.21	0.09	0.19	0.23	0.29
3	0.05	0.18	0.10	0.23	0.14	0.22	0.28	0.32
Main effect session	*F*_2, 3,476_ = 1.41, *p* = 0.24				*F*_2, 2,944_ = 4.66, *p* = 0.0095			
Main effect exercise	*F*_1, 16_ = 1.49, *p* = 0.24				*F*_1, 16_ = 9.96, *p* = 0.0061			
Exercise*session	*F*_2, 3,476_ = 0.78, *p* = 0.46				*F*_2, 2,944_ = 1.68, *p* = 0.186			
Latency to CR peak (ms)	455.83	169.76	451.14	150.79	466.63	176.32	495.63	131.48
Main effect exercise	*F*_1, 16_ = 0.002, *p* = 0.96				*F*_1, 16_ = 1.82, *p* = 0.20			
Main effect CR amplitude	*F*_1, 111_ = 16.03, *p* = 0.0001				*F*_1, 199_ = 9.58, *p* = 0.0022			
Percentage well-timed CRs	11.27	12.13	27.22	19.80	31.09	24.84	27.01	12.93
Main effect exercise	*F*_1, 16_ = 4.25, *p* = 0.056				*F*_1, 16_ = 0.19, *p* = 0.67			
**Spontaneous blinks (blinks/s)**								
1	0.30	0.22	0.22	0.22	0.21	0.27	0.48	0.37
2	0.30	0.24	0.26	0.23	0.26	0.25	0.50	0.43
3	0.24	0.22	0.23	0.17	0.32	0.27	0.44	0.35
Main effect session	*F*_2, 257_ = 1.06, *p* = 0.35				*F*_2, 229_ = 0.37, *p* = 0.69			
Main effect exercise	*F*_1, 15_ = 0.46, *p* = 0.51				*F*_1, 16_ = 2.79, *p* = 0.11			
Exercise*session	*F*_2, 257_ = 0.60, *p* = 0.55				*F*_2, 229_ = 2.14, *p* = 0.12			
UR amplitude	0.42	0.32	0.51	0.27	0.54	0.18	0.56	0.29
Main effect exercise	*F*_1, 14_ = 0.15, *p* = 0.71				*F*_1, 13_ = 0.11, *p* = 0.75			

All statistical comparisons done using an ANOVA on Linear Mixed-Effect Model. SD, standard deviation; CR, conditioned response; NEC, normalized eyelid closure; UR, unconditioned response.

In contrast, in the active group the CR amplitude differed between the no and post-exercise interventions (Figures 1D,F; Table 2). In the no exercise intervention, the mean CR amplitude at session 1 was 0.05 (±0.15) and increased to 0.14 (±0.22) by session 3. The mean CR amplitude for the post-exercise intervention was higher than the no exercise intervention at session 1 (mean = 0.18 ± 0.27), increasing to 0.28 (±0.32) by session 3. In the active group, the effect of “exercise” (*F*_1, 16_ = 9.96, *p* = 0.0061) and “session” (*F*_2, 2,944_ = 4.66, *p* = 0.0095) were significant. The interaction between “exercise” and “session” was not significant (*F*_2, 2,944_ = 1.68, *p* = 0.19). Post-hoc tests showed a significant difference between the active no and post-exercise interventions at sessions 1 (*t*_16_ = −2.73, *p* = 0.029) and 2 (*t*_16_ = −3.36, *p* = 0.012; Supplementary Table S1). In the active group with no exercise intervention, post-hoc tests showed a significant difference in CR amplitude between sessions 1 and 3 (*t*_2944_ = −1.98, *p* = 0.048). Likewise, in the active group with the exercise intervention, post-hoc tests showed a significant difference in CR amplitude between sessions 1 and 3 (*t*_2944_ = −2.39, *p* = 0.017).

### 3.4 Conditioning—timing

**Figure 2:**
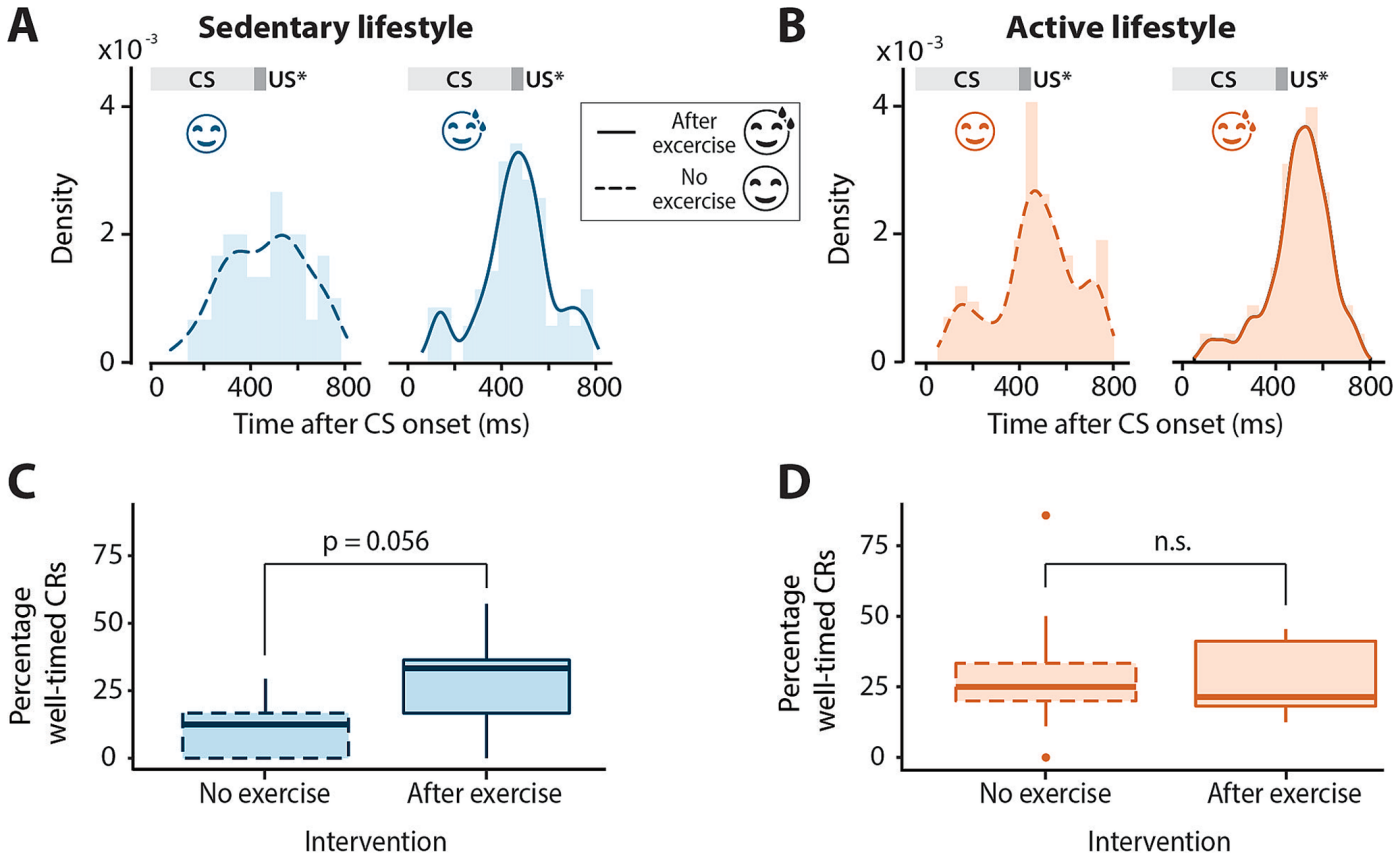
Timing of conditioned eyeblink responses in sedentary and active individuals with or without exercise. **(A)** Distribution of latency to conditioned response peak for all conditioned stimulus (CS) only trials across all sessions in sedentary and **(B)** active groups with or without exercise preceding eyeblink conditioning sessions. The dark gray block at 400 ms indicates the expected onset of the unconditioned stimulus (US*) which is omitted in these trials. The light gray block indicates the presentation of the CS. Note the distribution centered roughly around the expected onset of the US at 400 ms for all groups. **(C)** Boxplots of percentage of well-timed conditioned responses (CRs) in the sedentary and **(D)** active groups with or without exercise. Middle line indicates group medians, box ends indicate lower and upper quartiles, whiskers indicate group minima and maxima and dots indicate outliers. n.s. = not significant.

Next, it was determined whether acute aerobic exercise had an effect on the latency to CR peak. For this, CS-only trials were analyzed. For both lifestyle groups, the CR peak times are roughly distributed around the expected onset of the US regardless of the intervention (Figures 2A,B). Most of the variation in CR peak times could be explained by CR amplitude for both the sedentary (*F*_1, 111_ = 16.03, *p* = 0.0001) and active (*F*_1, 199_ = 9.58, *p* = 0.0022) lifestyle groups. The latencies to CR peaks did not differ significantly between no-exercise and post-exercise interventions for both sedentary (*F*_1, 16_ = 0.002, *p* = 0.96) and active (*F*_1, 16_ = 1.82, *p* = 0.20, Table 2) individuals.

The mean percentage of well-timed CRs was also determined for active and sedentary lifestyle groups with and without the exercise intervention (Table 2; Figures 2C,D). While the percentage of well-timed CRs was quite low for the sedentary group with no exercise intervention (Figure 2C, mean = 11.27% ±12.13), the effect of exercise on the percentage of well-timed CRs was neither significant for the sedentary (*F*_1, 16_ = 4.25, *p* = 0.056) nor the active group (*F*_1, 16_ = 0.19, *p* = 0.67).

### 3.5 Spontaneous blinks

**Figure 3:**
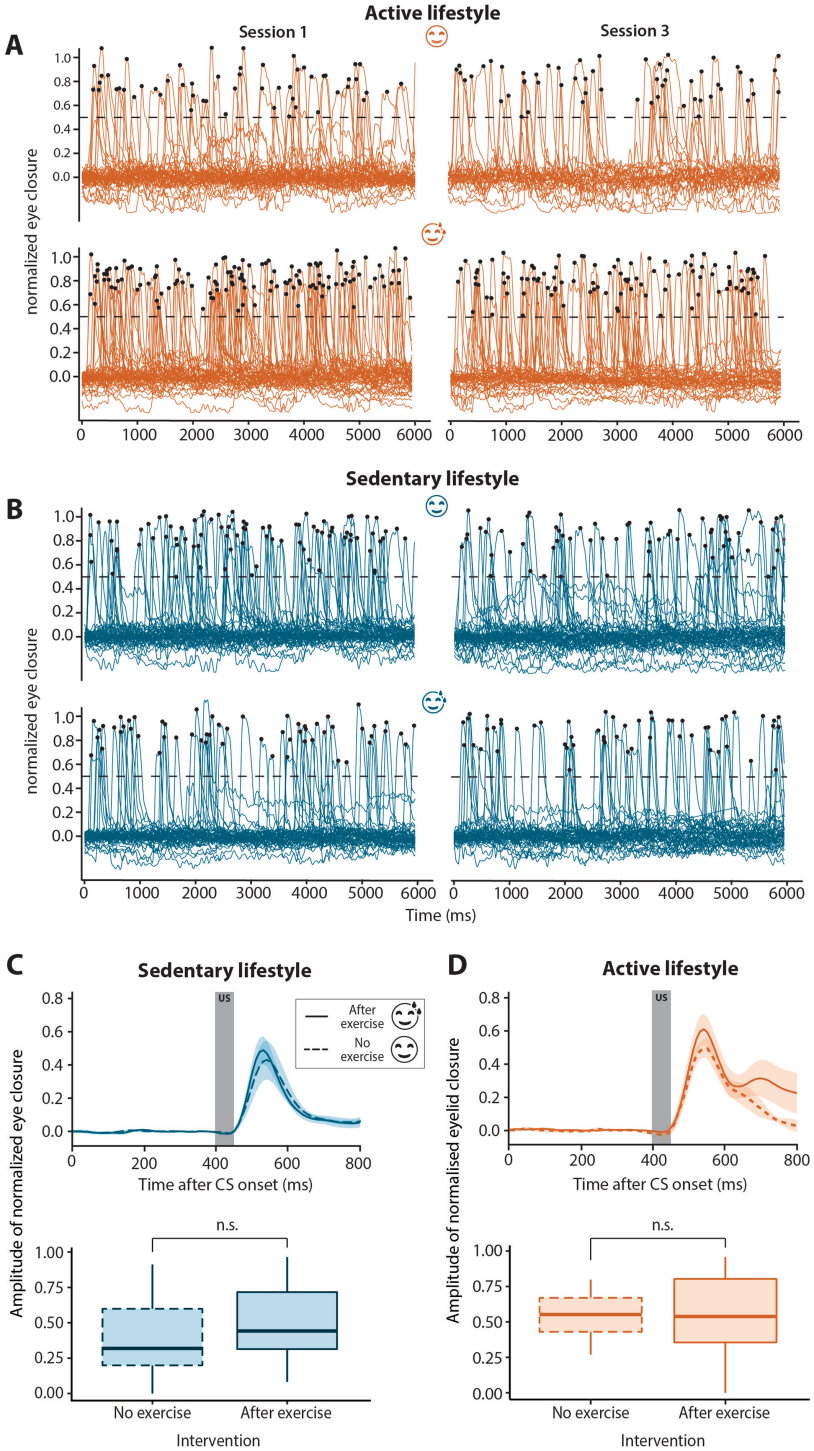
Spontaneous blinks and unconditioned stimulus auditory-evoked blinks in sedentary and active groups with or without exercise. **(A)** Spontaneous blinks for active no-exercise (**A**, top panel), active post-exercise (**A**, bottom panel), sedentary no-exercise (**B**, top panel) and sedentary post-exercise (**B**, bottom panel) interventions in sessions 1 (left-hand side) compared to session 3 (right-hand side). Spontaneous blinks were defined as peaks in the eyelid signal data with an amplitude >0.5 indicated by the dotted black line. Black dots indicate the peaks of traces classified as spontaneous blinks. Eyeblink traces for all six “dummy” trials per session for all participants are shown. The number of spontaneous blinks per second did not change significantly over the course of conditioning for any of the interventions for either active or sedentary individuals. **(C)** Group averaged unconditioned response amplitudes for sedentary or **(D)** active individuals with (solid line) or without (dashed line) exercise preceding the eyeblink conditioning session. Unconditioned response amplitudes were calculated for the first two blocks of session 1, prior to the development of conditioned responses. The dark gray block (top panels) from 400 to 450 ms indicates the presentation of the unconditioned stimulus (US). The unconditioned response amplitude was similar regardless of the exercise intervention for both sedentary and active groups. In the boxplots (bottom panels), the middle line indicates group medians, box ends indicate lower and upper quartiles, whiskers indicate group minima and maxima. n.s. = not significant.

To assess whether the observed differences in CR amplitude were indeed representative of associative learning and not due to non-associative sensitization, we analyzed the number of spontaneous blinks over the course of conditioning in active and sedentary groups with and without exercise prior to eyeblink conditioning (Figures 3A,B). The mean number of spontaneous blinks are reported in Table 2 and did not differ between the no and after exercise interventions for both active and sedentary individuals. There was no main effect of exercise or session and no session*exercise interaction effect on the number of spontaneous blinks.

### 3.6 Unconditioned responses

To determine if the effect of aerobic exercise was specific to CRs or more generalized, unconditioned response amplitude was compared between interventions within lifestyle groups. The block 1 trials of session 1 were used to determine unconditioned response amplitude, as these data were obtained before onset of the CRs that could in principle influence the amplitude of the unconditioned response (Green et al., 2011). In both the sedentary (*F*_1, 14_ = 0.15, *p* = 0.71) and active groups (*F*_1, 13_ = 0.11, *p* = 0.75), there was no significant difference in unconditioned response amplitude between the no and post-exercise interventions (Figures 3C,D; Table 2).

## 4 Discussion

Aerobic exercise enhanced CR acquisition in a smartphone-mediated eyeblink conditioning paradigm. This effect of exercise was, however, only seen in individuals with an active lifestyle. This finding parallels that of Hopkins and colleagues (Hopkins et al., 2012), where acute exercise enhanced recognition memory in individuals with a prior 4 week exercise training program, but not in individuals without such a program. Exercise had no major effect on the unconditioned response amplitude, number of non-associative spontaneous blinks or the timing of CR peaks.

### 4.1 Conditioned response acquisition

Both the active post-exercise and no-exercise interventions showed a significant increase in CR amplitude over the three sessions. The significant differences in CR amplitude between the active no-exercise and active post-exercise groups at early sessions parallels the acute exercise enhancing effect seen in animal research where mice running at faster speeds showed CRs in earlier sessions compared to mice running at slower speeds (Albergaria et al., 2018). Exercise may have a priming effect; reducing the number of practice sessions needed for implicit learning (Mang et al., 1985). This enhancing effect of exercise was specific to CRs as the amplitude of the unconditioned responses did not differ between groups. It is proposed that locomotor activity acts directly within the cerebellar cortex to modulate eyeblink conditioning. Locomotor activity signaling via cerebellar mossy fibers (MF) may converge with the CS MF signaling hereby facilitating learning (Albergaria et al., 2018). While exercise may have acted directly within the cerebellar cortex to enhance associative learning (Broersen et al., 2023), it is unclear why such an effect would differ for active and sedentary individuals.

The finding that acute exercise facilitates eyeblink conditioning in active but not sedentary individuals may point toward a mechanistic role of neuropeptidergic transmitters and/or neurotrophins. Indeed, both human (Hopkins et al., 2012; Nofuji et al., 2012; Szuhany et al., 2015) and animal (Berchtold et al., 2005) studies on neuropeptidergic transmitters and neurotrophins show differential effects of acute exercise in active compared to sedentary subjects. Likewise, the dopaminergic, adrenergic and norepinephrinergic pathways, which are all catecholaminergic systems that prominently co-release neuropeptides, are upregulated in humans (Winter et al., 2007; Skriver et al., 2014) and animals (Chaouloff, 1989; Meeusen et al., 2001; Pagliari and Peyrin, 1995) following exercise. While the proposed role of these neurotransmitters in exercise-induced cognitive benefits are frequently studied (Winter et al., 2007; McMorris, 2016), their potential influence on associative learning has received less attention (Skriver et al., 2014; Flace et al., 2021). Despite this, there is evidence for a role of neurotransmitters in cerebellar learning. In rabbits, pharmacological monoamine depletion resulted in a dose-dependent reduction in CRs in an eyeblink conditioning task (Winsky and Harvey, 1992). Additionally, in rats, cerebellar norepinephrine was shown to be involved in the acquisition of CRs (Cartford et al., 2004; Paredes et al., 2009). These findings may extend to humans, where increased levels of norepinephrine following exercise have been associated with improved motor skill acquisition compared to resting controls (Skriver et al., 2014) and where chronic training increased the plasma catecholamine response, compared to no training, after a cycling task (Silverman and Mazzeo, 1996).

Similarly, the neurotrophin BDNF may facilitate exercise-induced brain plasticity (Berchtold et al., 2005; Walsh and Tschakovsky, 2018; Neeper et al., 1995) and memory formation (Basso and Suzuki, 2017; Camuso et al., 2022). Notably, BDNF mutant mice show impaired eyeblink conditioning (Qiao et al., 1998) and the impact of tDCS on eyeblink conditioning in humans can depend on BDNF mutations (van der Vliet et al., 2018). Moreover, a meta-analysis of the effects of exercise on BDNF in humans reported an enhanced BDNF response to acute exercise in active compared to sedentary individuals (Szuhany et al., 2015). Thus it is possible that in this study the acute exercise in the sedentary group was not sufficient to induce the BDNF levels needed to enhance eyeblink conditioning. Together, these findings provide tentative molecular clues as to why in this study aerobic exercise enhanced associative learning in active but not sedentary individuals.

### 4.2 Conditioned response timing

Unlike the acquisition of CRs, the timing of these responses did not significantly differ across groups. Similarly, CR timing was unaltered in rats with access to a running wheel despite these rats showing enhanced CRs compared to non-exercising rats (Green et al., 2011). Interestingly, while not significantly different, the percentage of well-timed CRs was slightly higher in the sedentary post-exercise compared to the sedentary no-exercise group. This is in-line with a recent study where physically enriched mice showed more well-timed CRs compared to mice in standard environments (Dijkhuizen et al., 2024) and may require further research in a larger sample.

### 4.3 Limitations and future work

This study was limited by a lack of detailed information on more general physical activity levels in the groups. The study specifically focused on exercise and so no data was collected on other forms of physical activity. Understanding how physical activity may influence the effect of exercise on eyeblink conditioning was, however, beyond the scope of this study and would be of interest to investigate in future work.

Despite all participants being instructed to exercise at moderate intensity, the average self-reported exercise intensity rating differed between active and sedentary individuals, with sedentary individuals perceiving the exercise to be harder than active individuals. While not directly comparable to a physical stressor, a psychosocial stressor impaired eyeblink conditioning in human subjects (Wolf et al., 2009). The increased perceived intensity of exercise in the sedentary group may have masked the exercise enhancing effects seen in the active group. Interestingly, however, the average heart rate during the intervention was not significantly different between lifestyle groups, although not all participants recorded their heart rate. Future work could further explore potential associations between exercise intensity and eyeblink conditioning using objective as well as subjective measures of exercise intensity.

An additional limitation of this study was the lack of power to analyze sex differences between groups. A sex-dependency in eyeblink conditioning performance has been shown, with females showing more CRs compared to males (Löwgren et al., 2017). The generalizability of the findings are limited as the study was conducted on mainly white participants with middle to high socio-economic status. As this was not a randomized controlled trial (RCT), direct causal relationships between exercise and associative learning cannot be made. A future RCT could assess the level of exercise training required before acute effects of exercise on learning are seen.

Finally, future directions could include investigating a possible clinical application of these findings. Studies have shown impaired eyeblink conditioning in various patient groups, for example schizophrenia (Brown et al., 2005), ADHD (Frings et al., 2010) and spinocerebellar ataxia (Timmann et al., 2005). Whether exercise could restore eyeblink conditioning deficits in these patient groups and hereby inform exercise-based therapeutic interventions remains to be determined.

## 5 Conclusion

Acute aerobic exercise enhanced the acquisition of associative learning in an eyeblink conditioning paradigm for individuals engaging in regular exercise. These results tentatively confirm in humans what has been shown in animals regarding the facilitatory effects of exercise on eyeblink conditioning. By focusing on a well-characterized learning paradigm, this study contributes to a more objective understanding of how exercise influences the brain, however, given the exploratory nature of this study, replicating the results in a larger cohort will be an important next step.

## Data Availability

The raw data supporting the conclusions of this article will be made available by the authors, without undue reservation.
