# Supplementary material for: Acute aerobic exercise enhances associative learning in regular exercisers but not in non-regular exercisers
Source: Front Behav Neurosci. 2025 Jan 7;18:1515682. doi: 10.3389/fnbeh.2024.1515682 (PMC11747211; doi:10.3389/fnbeh.2024.1515682)
Supplement: Supplementary file 1 [file Table_1.docx]

Supplementary Material

# Supplementary Figures and Tables

## Supplementary Figures


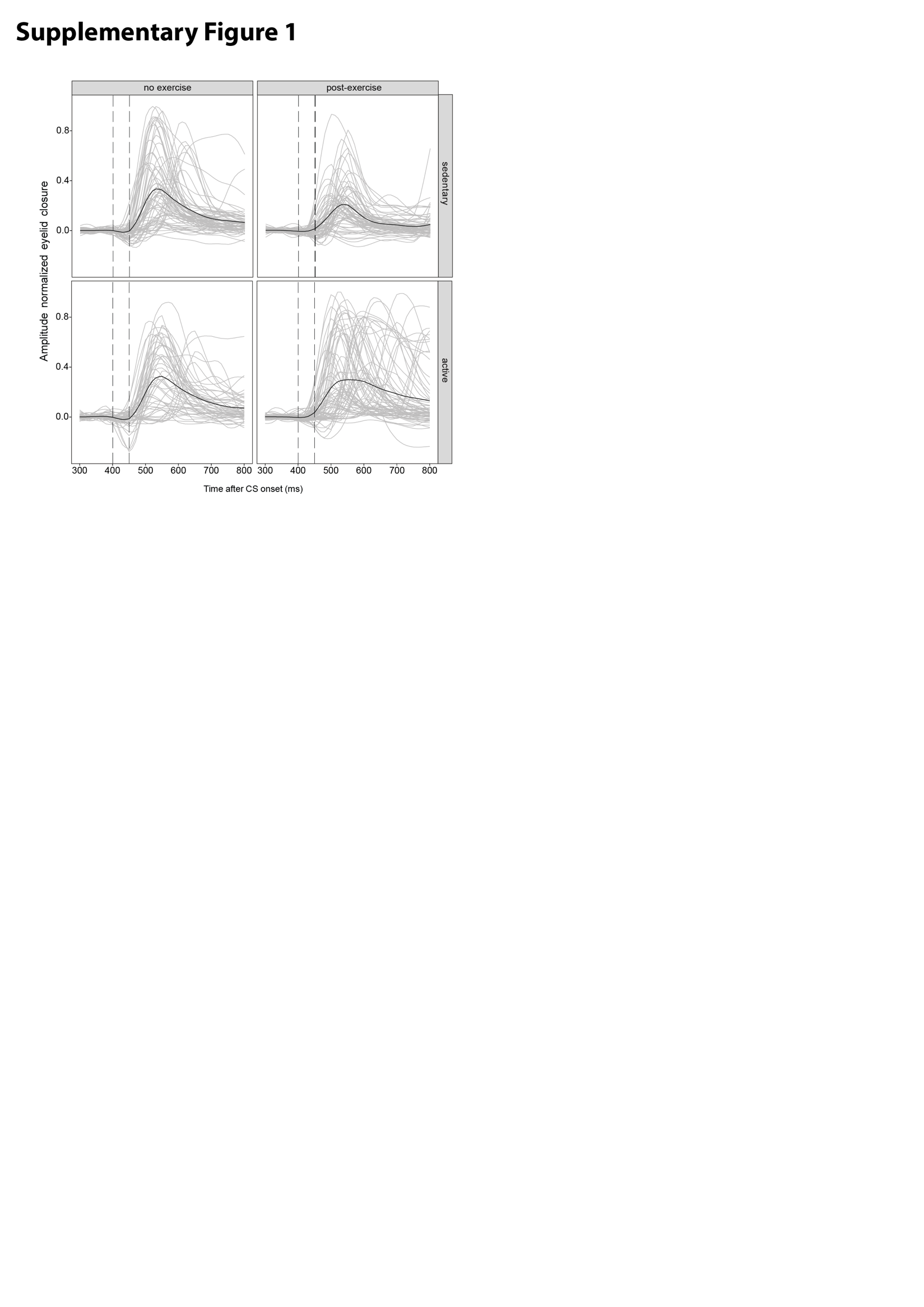


**Supplementary Figure 1.** **Raw traces of auditory evoked blinks for sedentary and active groups with or without an exercise intervention.** Unconditioned stimulus only trials from session 1 in sedentary and active individuals without (left panels) and directly after (right panels) an exercise intervention. Grey traces represent subject averages and black traces represent group averages. The first vertical dashed line represents the onset of the unconditioned stimulus at 400 ms and the second dashed line represents the offset of the unconditioned stimulus at 450 ms. Note the latency in response to presenting the unconditioned stimulus in all groups.


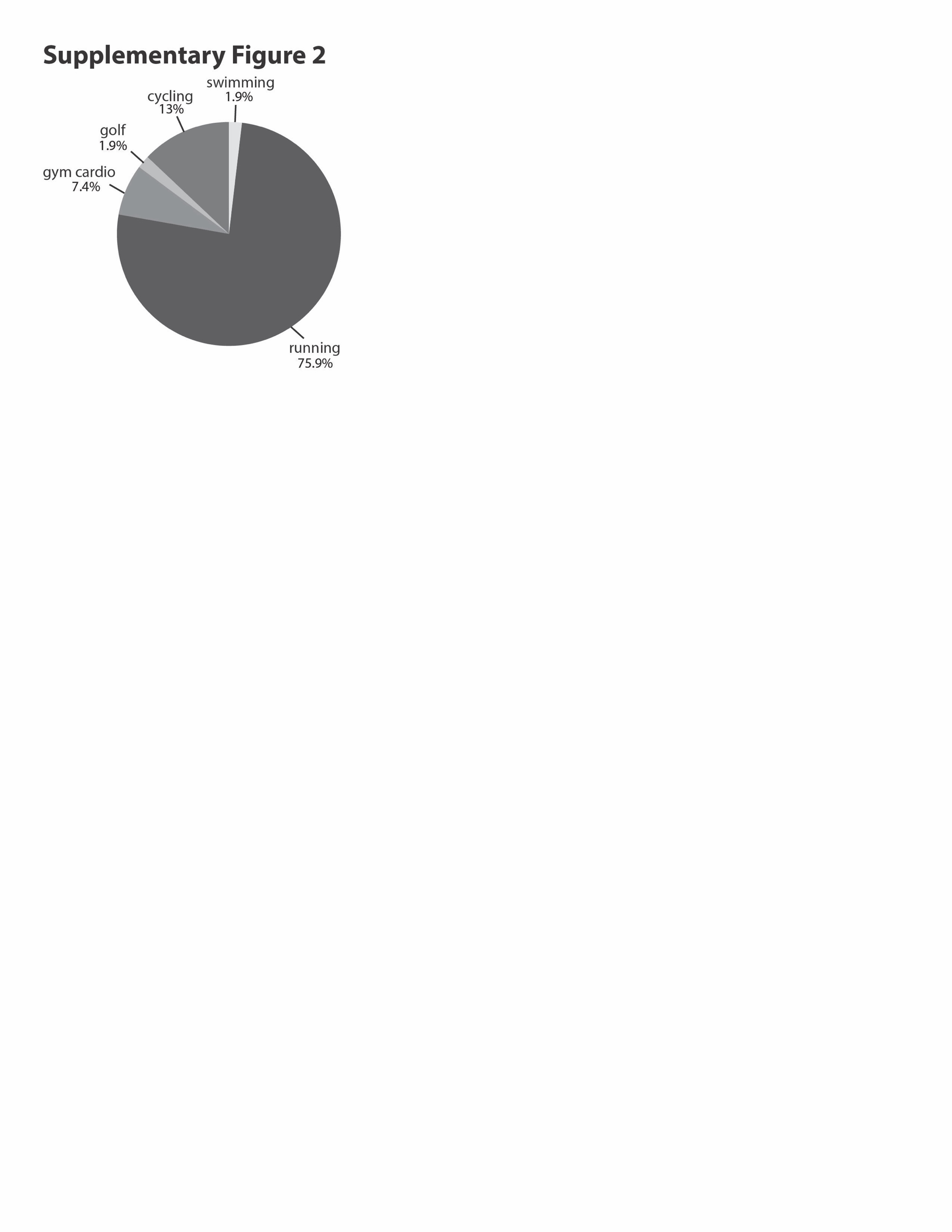


**Supplementary Figure 2. Frequency of types of exercise done prior to eyeblink conditioning in the exercise intervention.** Distribution of exercise activities done by active and sedentary individuals in the exercise intervention. Distribution includes all three exercise sessions per participant done prior to the three eyeblink conditioning sessions


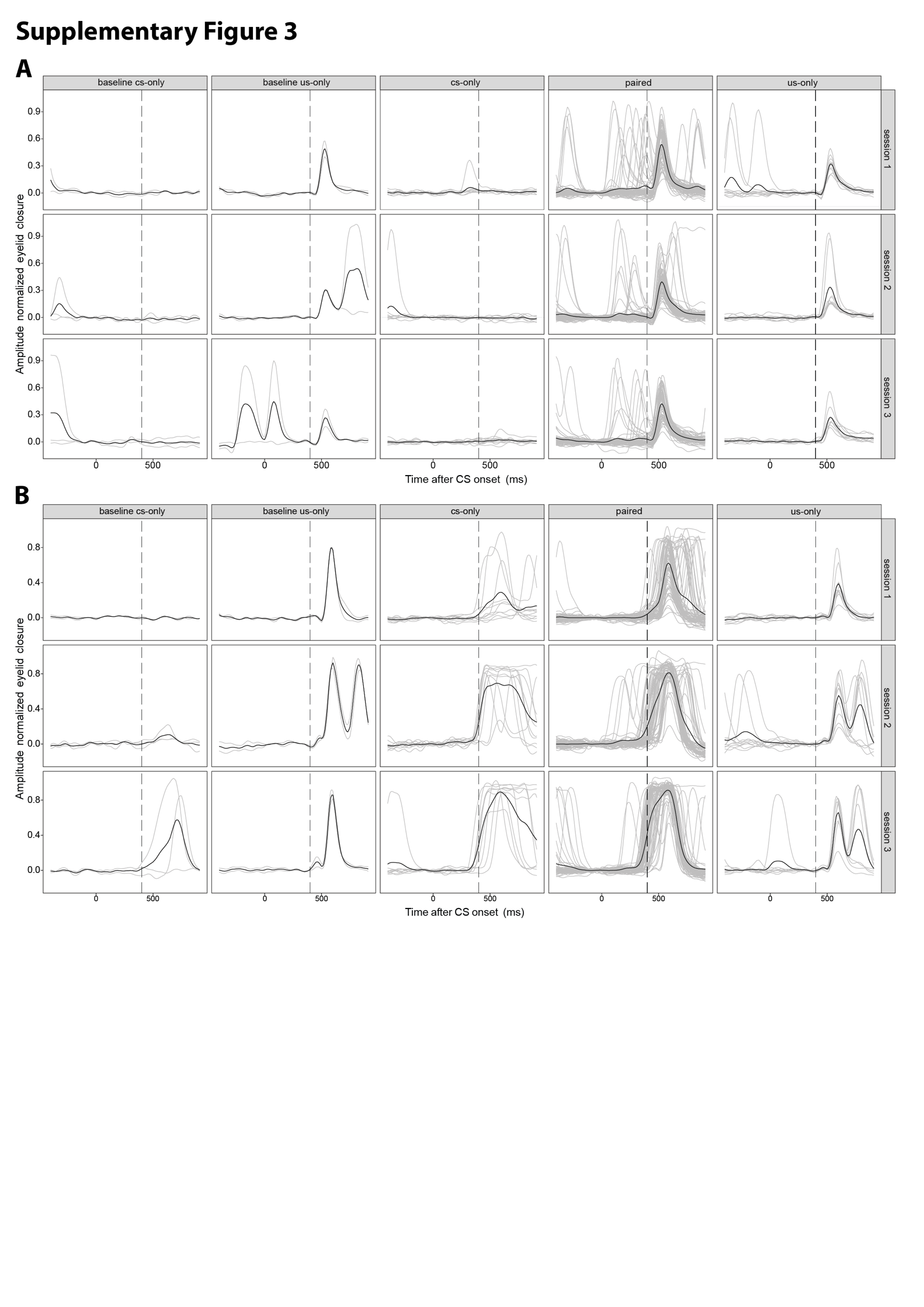


**Supplemental Figure 3** | **Raw eyeblink traces from two participants for all trial types.** Example traces of a poor learner, from the sedentary no-exercise intervention **(A)** and a learner from the active post-exercise intervention **(B)** for all trials over 3 sessions of delay eyeblink conditioning. Vertical line at 400 ms showing expected (conditioned stimulus (CS) -only trials) or actual presentation of the unconditioned stimulus (US). Gray traces indicate trials, black traces indicate session averages. Numbers on the right-hand side indicate session numbers.

## Supplementary Table

**Supplemental Table 1 | Post-hoc tests within sedentary and active groups for no vs. post-exercise interventions prior to three sessions of eyeblink conditioning** All statistical comparisons done using an ANOVA on Linear Mixed-Effect Model

P-value adjustment: Bonferroni-Holm method for three tests for comparisons between interventions

|  | **Pairwise differences** | | | |
| --- | --- | --- | --- | --- |
|  | **estimate** | **t-ratio** | **p-value** | **adjusted p-value** |
| **Sedentary** |  | | | |
| **Session 1: no vs. post-exercise** | -0.19 | -0.98 | 0.34 | 0.68 |
| **Session 2: no vs. post-exercise** | -0.28 | -1.43 | 0.17 | 0.52 |
| **Session 3: no vs. post-exercise** | -0.22 | -0.98 | 0.34 | 0.68 |
| **Sedentary no exercise: session 1 vs. session 3** | -0.14 | -1.12 | 0.26 | 0.26 |
| **Sedentary post-exercise: session 1 vs. session 3** | -0.16 | -1.30 | 0.20 | 0.20 |
| **Active** |  | | | |
| **Session 1: no vs. post-exercise** | -0.44 | -2.73 | 0.014 | 0.029 |
| **Session 2: no vs. post-exercise** | -0.60 | -3.36 | 0.0040 | 0.012 |
| **Session 3: no vs. post-exercise** | -0.48 | -1.99 | 0.064 | 0.064 |
| **Active no exercise: session 1 vs. session 3** | -0.31 | -1.98 | 0.048 | 0.048 |
| **Active post-exercise: session 1 vs. session 3** | -0.35 | -2.39 | 0.017 | 0.017 |
